# Serum Lithium Concentration and the Risk of Chronic Kidney Disease

**DOI:** 10.1192/j.eurpsy.2024.529

**Published:** 2024-08-27

**Authors:** G. Gislason, Ó. S. Indriðason, R. Pálsson, E. Sigurðsson

**Affiliations:** ^1^Faculty of Medicine, University of Iceland; ^2^Division of Nephrology; ^3^Mental Health Services, Landspitali–The National University Hospital of Iceland, Reykjavík, Iceland

## Abstract

**Introduction:**

Lithium is an important treatment option for individuals with mood disorders, but its use has been linked to the development of chronic kidney disease (CKD). Existing studies on this association have reported conflicting results.

**Objectives:**

The aim of this study was to examine the risk of developing CKD with lithium use adjusting for common comorbidities.

**Methods:**

This was a retrospective cohort study that included all individuals in Iceland receiving lithium therapy between 2008 and 2018. Lithium use was defined as at least one dispensed prescription for Lithium or at least one serum lithium concentration above the detection limit. Patients with affective disorders (ICD-10 codes F30-F39) attending the outpatient clinics of Landspitali–The National University Hospital Mental Health Services in 2014-2016, without lithium exposure, served as controls. CKD stages 3-5 were defined according to the Kidney Disease Improving Global Outcomes (KDIGO) guidelines for CKD as estimated glomerular filtration rate (eGFR) less than 60 mL/min/1.73 m^2^. The eGFR was calculated using the serum creatinine (SCr) based on the *Chronic Kidney Disease Epidemiology Collaboration* (CKD-EPI) equation. Acute kidney injury (AKI) was defined according to the SCr component of the KDIGO criteria for AKI, and other comorbid diseases were defined based on ICD-9 and ICD-10 codes. Individuals with fewer than 2 SCr measurements during the study period and those with CKD stages 3-5 prior to 2008 were excluded. Cox regression analysis with time dependent variables was performed to assess the risk of CKD.

**Results:**

The study included 2046 individuals exposed to lithium, of whom 221 (10.9%) developed CKD in the study period. Among the 1220 control subjects, 39 (3.2%) developed CKD. Lithium use was associated with CKD (hazard ratio [HR] 1.93, 95% confidence interval [CI] 1.37–2.74) after adjusting for sex, age, and comorbid diseases. Other significant risk factors were age (per year, HR 1.03, 95% CI 1.02–1.04), initial eGFR (per mL/min/1.73 m^2^, HR 0.92-0.96, 95% CI 0.90–0.99), presence of diabetes (HR 1.73, 95% CI 1.15–2.48) and history of AKI (HR 1.89, 95% CI 1.32–2.70). When compared to the control group not exposed to lithium, the risk (HR) of CKD was 1.24 (95% CI 0.81–1.89), 2.88 (95% CI 1.97–4.20) and 5.23 (95% CI 3.31-8.26) for groups with a mean lithium concentration of 0.3-0.59, 0.6-0.79 and 0.8-0.99 mmol/L, respectively.

**Conclusions:**

Long-term lithium therapy seems to increase the risk of CKD in a concentration-dependent manner in individuals with bipolar and unipolar mood disorders. To mitigate this risk, it is essential to monitor blood levels carefully and use doses of lithium as low as possible for adequate mood stabilization and treatment.

**Disclosure of Interest:**

None Declared

